# Membrane Vesicles Improve *Streptococcus mutans* Early Biofilm Formation

**DOI:** 10.3390/microorganisms14040826

**Published:** 2026-04-03

**Authors:** Yina Cao, Yue Li, Yinghong Zhou

**Affiliations:** 1 Hospital of Stomatology, Guanghua School of Stomatology, Sun Yat-Sen University, Guangzhou 510055, China; 2 Guangdong Provincial Key Laboratory of Stomatology, Sun Yat-Sen University, Guangzhou 510055, China; 3 School of Dentistry, Centre for Orofacial Regeneration, Reconstruction and Rehabilitation (COR3), The University of Queensland, Brisbane 4006, Australia

**Keywords:** *Streptococcus mutans*, membrane vesicles, biofilm formation

## Abstract

*Streptococcus mutans* (*S. mutans*), one of the main etiological pathogens of dental caries, forms dental plaque biofilms that drive tooth decay. Although bacterial membrane vesicles (MVs) are increasingly recognized as modulators of biofilm biology, little is known about MVs generated by *S. mutans*. The objective of this study is to investigate the role of *S. mutans*-derived MVs in the development of *S. mutans* biofilms formed under static conditions in plates or confocal dishes. Transmission electron microscopy and nanoparticle tracking analysis revealed that the MVs were cup-shaped with bilayered membranes and averaged 80.49 ± 32.24 nm in diameter. The addition of ≥5 µg/mL MVs enhanced biofilm formation during the initial adhesion stage (0 to 6 h), as demonstrated by crystal violet staining and XTT assays. Confocal laser scanning microscopy and scanning electron microscopy confirmed the incorporation of PKH26-labeled MVs into *S. mutans* biofilms and showed that supplemental MVs increased bacterial viability and extracellular polysaccharide biomass. Furthermore, RT-qPCR analysis revealed upregulated expression of genes related to adhesion and quorum-sensing systems in MV-treated biofilms. In conclusion, these findings indicate that *S. muants* MVs are integral biofilm components that promote biofilm establishment at the early stage of biofilm formation.

## 1. Introduction

According to The Lancet (2017), dental caries is a major public health issue in the world [1]. *Streptococcus mutans* (*S. mutans*), a Gram-positive bacterium, is considered one of the primary pathogens responsible for dental caries [2]. Biofilm formation is an important virulence trait of *S. mutans* [2,3], as biofilms represent the predominant state of oral microorganisms on tooth surfaces. Through biofilm formation, *S. mutans* can recruit bacterial cells and persist against antimicrobial challenges.

Although bacterial vesicles were first reported in the 1960s, it was not until the 1990s that Gram-positive bacteria were shown to produce membrane vesicles (MVs) [4]. These vesicles, typically 20–400 nm in diameter, contain nucleic acids, lipids, proteins and metabolites [5]. Research on Gram-negative bacterial MVs has advanced rapidly in recent decades, whereas studies on Gram-positive bacterial MVs remain limited, likely due to their thicker cell walls and lower vesicle yields [6]. MVs are now recognized as mediators of cell-free intercellular communication across all domains of life. Several studies have demonstrated their involvement in biofilm formation and stability [7,8,9]. For example, MVs have been identified in the biofilm matrix of *Bacillus subtilis* [7] and within the extracellular matrix surrounding the outer portion of *Mycobacterium ulcerans* biofilms [9]. In addition, MVs isolated from planktonic *Listeria monocytogenes* were shown to contain protein components associated with biofilms [10].

Despite increasing evidence linking MVs to biofilm development [11,12,13,14], little is known about MVs generated by *S. mutans*. In 2014, Liao reported that *S. mutans* MVs can serve as a vehicle for extracellular DNA release [15]. In 2019, Ohnishi and colleagues reported that glucosyltransferases (Gtfs) carried by MVs enhance biofilm formation. Our own proteomic analysis of *S. mutans* MVs (unpublished) revealed multiple MV-associated proteins involved in adhesion. These findings suggest that *S. mutans* MVs play an important role in the formation of biofilm. However, the regulatory mechanisms underlying their contribution remain poorly understood.

The aim of this study is to investigate the role of *S. mutans*-derived MVs in the development of *S. mutans* biofilms formed under static conditions in plates or confocal dishes. This work will expand current knowledge of *S. mutans* MVs and provide insights into their potential role in the pathogenesis of dental caries.

## 2. Materials and Methods

### 2.1. Bacterial Strain and Growth Conditions

*S. mutans* UA159 (ATCC700610) was utilized in the study and grown in brain–heart infusion (BHI, Difco, Detroit, MI, USA) broth. For biofilm formation, overnight cultures were inoculated into 96-well flat-bottom plates containing 1% BHIS (BHI supplemented with 1% [wt/vol] sucrose). Cultures were incubated under anaerobic conditions (5% CO_2_, 10%H_2_, and 85% N_2_) at 37 °C [16,17,18,19,20].

### 2.2. Isolation and Purification of MVs

MVs were prepared from *S. mutans* cultures as described previously, with minor modifications [15]. Briefly, 1 L of overnight *S. mutans* cultures was centrifuged at 6000× *g* for 15 min at 4 °C, followed by 10,000× *g* for 15 min at 4 °C. The supernatant was collected and filtered through a 0.22 μm pore-size membrane filter (Millipore, Tullagreen, Ireland) to remove residual cells. The filtrate was concentrated using a 100 kDa cutoff tangential flow filter (Millipore, Tullagreen, Ireland) and then subjected to ultracentrifugation at 100,000× *g* for 70 min at 4 °C (Beckman Coulter, Brea, CA, USA). The resulting MV pellet was resuspended in PBS and stored at −80 °C for further research.

MV morphology was confirmed using transmission electron microscopy (TEM) [11]. Briefly, 10 μL of purified MVs was adsorbed onto a carbon-coated grid, negatively stained with 3% uranyl acetate (Guangzhou Chemical Reagent Factory, Guangzhou, China), rinsed with double-distilled water to remove excess stain, air-dried at room temperature, and visualized with a Hitachi TEM System HC-1 (Hitachi, Tokyo, Japan) at 80 KV. The size distribution of MVs was determined by Nanoparticle Tracking Analysis (NTA) using a ZetaView particle tracker (Particle Metrix, Inning am Ammersee, Germany) [21]. The yield of MVs was quantified by measuring protein concentration using a BCA Protein Assay Kit (CWbio, Beijing, China).

### 2.3. Confocal Laser Scanning Microscopy (CLSM)

#### 2.3.1. Observation of PKH26-Labeled MVs

MVs were labeled with PKH26 (Sigma, St. Louis, MO, USA), according to the manufacturer’s protocol, with minor modifications [22,23]. Labeled MVs (5 μg/mL) were added to *S. mutans* cultures in 1% BHIS within 15 mm confocal dishes. PBS was used as a control. After 24 h of incubation, PKH26 fluorescence was visualized using CLSM with excitation at 633 nm [23].

#### 2.3.2. Live/Dead Bacterial Staining

To evaluate bacterial viability, *S. mutans* biofilms were cultured in 1 mL of 1% BHIS with either PBS or 5 μg/mL MVs in 15 mm confocal dishes and incubated for 24 h. Planktonic cells were removed by washing with PBS, and the biofilms were stained with the LIVE/DEAD BacLight Bacterial Viability Kits (Molecular Probes, Eugene, OR, USA) for 15 min. CLSM detection was performed at 543 nm for SYTO9 and 488 nm for propidium iodide (PI). Images were obtained and analyzed using Image J version 1.54p with the COMSTAT plugin version 2 [24].

#### 2.3.3. Extracellular Polysaccharide (EPS) Analysis

For EPS visualization, *S. mutans* was cultured in 1 mL of 1% BHIS containing 5 μg/mL MVs and 1 μM Alexa Fluor 647 (Invitrogen, Carlsbad, CA, USA) in 15 mm confocal dishes. Cultures were protected from light and incubated for 24 h, with PBS as a negative control. After incubation, biofilms were gently washed with normal saline and counterstained with 1 μM SYTO9 at room temperature for 15 min to label live bacteria. CLSM was performed with detection set at 655–690 nm for Alexa Fluor 647 and 495–515 nm for SYTO9. Images were obtained and analyzed using Image J version 1.54p with the COMSTAT plugin version 2 [25].

For CLSM observation, three replicate dishes were prepared per condition in each experiment, with at least five randomly selected microscopic fields imaged per dish. All experiments were performed independently three times.

### 2.4. Crystal Violet (CV) Assay

Overnight cultures of *S. mutans* were inoculated into 96-well plates containing 1% BHIS, and purified MVs were added in a dose-dependent manner to final protein concentrations of 1, 2, 5, 10, 15 and 20 μg/mL. An equal volume of PBS was used as a control. Plates were incubated at 37 °C for 24 h under static conditions to allow biofilm formation. Biofilm quantitation was carried out using the crystal violet (CV) assay [26]. Briefly, after incubation, cultures were removed, and the wells were washed with PBS, fixed with 95% methanol (Thermo Fisher Scientific, Pittsburgh, PA, USA), washed again, and stained with 0.1% (wt/vol) CV (Sigma-Aldrich, St. Louis, MO, USA) solution for 15 min at room temperature. Excess stain was removed by washing, and the retained CV was dissolved in 100 μL of 100% ethanol (Guangzhou Chemical Reagent Factory, Guangzhou, China) for 15 min at room temperature. Absorbance was measured at 600 nm using a spectrophotometer. To assess the time-dependent effect of MVs, 5 μg/mL MVs were added to *S. mutans* cultures in 1% BHIs at 0, 6, or 12 h during a 24 h biofilm growth period, and biofilm biomass was quantified using the CV assay. All experiments were carried out in triplicate and repeated independently three times.

### 2.5. XTT Assay

Biofilm metabolic activity was assessed at 24 h using the XTT assay [27]. Briefly, biofilms grown in 96-well plates were washed with PBS twice, and 200 µL of XTT–menadione solution (180 mg/L XTT combined with 0.688 mg/L menadione; Sigma-Aldrich, St. Louis, MO, USA) was added to each well. Plates were incubated for 3 h at 37 °C with gentle shaking. Following incubation, 100 µL of supernatant from each well was transferred to a fresh 96-well plate, and absorbance was measured at 492 nm using a spectrophotometer. We used 3 technical replicates per condition in each of the 3 independent biological experiments.

### 2.6. Scanning Electron Microscopy (SEM)

Biofilm morphology was examined using SEM. (Quanta 400F, FEI/Thermo Fisher Scientific, Eindhoven, The Netherlands) Sterile glass coverslips were placed in 12-well plates, and biofilms were allowed to form by culturing *S. mutans* in 1% BHIS with or without the addition of 5 µg/mL MVs. PBS was used as a negative control. After 24 h of incubation at 37 °C, coverslips were gently washed to remove planktonic cells and fixed overnight at 4 °C with 2.5% glutaraldehyde (Zhongjing Keyi, Kaifeng, China) in PBS. Samples were dehydrated through a graded ethanol series (30%, 50%, 70%, 80%, 85%, 90%, 95%, and 100%; Guangzhou Chemical Reagent Factory, Guangzhou, China), dried in a desiccator, and sputter-coated with gold. Biofilms were visualized using SEM at magnifications of 2000× and 5000× [25,28].

### 2.7. Gene Expression Analysis by Real-Time PCR

Biofilms were grown for 24 h and treated with either MVs or PBS (control). Biofilms were harvested by centrifugation at 12,000 rpm for 5 min, and total RNA was extracted using ultrasonic disruption followed by purification with the RNeasy Mini Kit (QIAGEN, Valencia, CA, USA). RNA concentration and purity were determined using a NanoDrop 2000 spectrophotometer (Thermo Fisher Scientific, Pittsburgh, PA, USA). cDNA synthesis and quantitative real-time PCR (qRT-PCR) were performed as previously described. Primer sequences were obtained from the literature and are listed in Table 1 (primer sequence, 5′-3′). Relative gene expression levels were calculated using the 2^−ΔΔCt^ method, with normalization to internal reference genes [25].

### 2.8. Statistical Analysis

All experiments were performed at least three times independently. Data were initially analyzed using conventional statistical methods (unpaired *t*-test for two groups and one-way ANOVA with Tukey’s post hoc test for multiple groups) in GraphPad Prism 5.04. To further validate the robustness of the findings, representative datasets (corresponding to Figures 2a,b and 5b) were additionally re-analyzed using a linear mixed-effects model with experiment as a random effect and treatment as a fixed effect (GraphPad Prism 8.0.2). The mixed-effects model results confirmed the conclusions obtained with the conventional methods. Full details of the mixed-effects model analyses are provided in the Supplementary Materials (Figures S1–S3). A *p*-value < 0.05 was considered statistically significant.

## 3. Results

### 3.1. Morphology of MVs Isolated from BHI Broth

**Figure 1:**
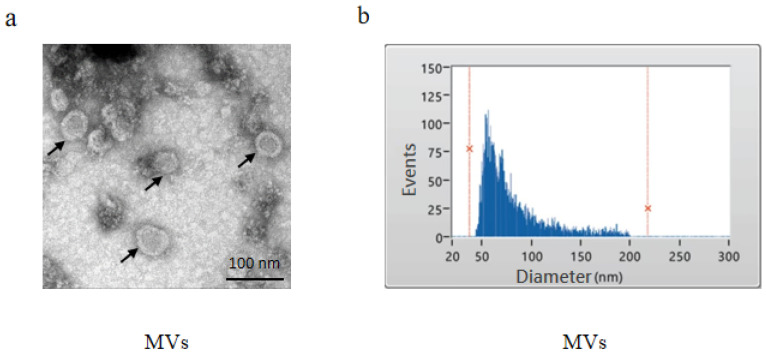
Identification of *S. mutans* MVs. (**a**) TEM showing the morphology of MVs with bilayered membranes. Arrows indicate representative MVs. (**b**) NTA showing the size distribution of MVs, with an average diameter of 80.49 ± 32.24 nm. The red vertical line and red ‘x’ represent the analysis boundaries. The left red cross marks the minimum detection size, and the right red cross marks the maximum detection size.

*S. mutans* was cultured in BHI broth overnight to the stationary phase, and MVs were subsequently isolated from the culture supernatant. TEM revealed that the MVs possessed bilayered membranes and were approximately 100 nm in diameter (Figure 1a). The NTA assay further confirmed the size distribution, showing an average MV diameter of 80.49 ± 32.24 nm (Figure 1b).

### 3.2. MVs Improve Biofilm Formation During the Initial Adhesion Stage

**Figure 2:**
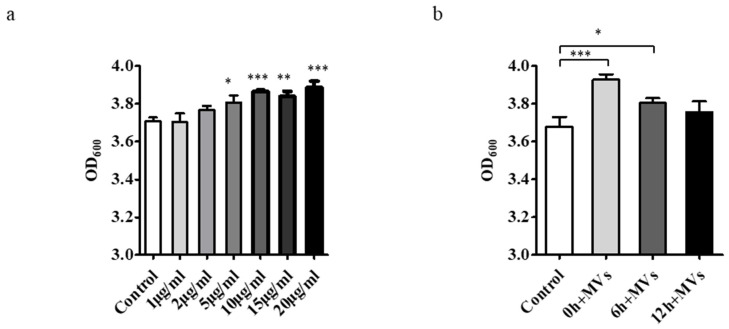
Crystal violet assay of *S. mutans* biofilm formation at the initial adhesion stage. (**a**) *S. mutans* was cultured in 1% BHI with increasing concentrations of MVs (0, 1, 2, 5, 10, 15, 20 µg/mL). Biofilm biomass was quantified using the crystal violet assay at 600 nm. (**b**) MVs (5 µg/mL) were added at 0, 6, or 12 h during biofilm development in 1% BHIs, and biofilm biomass was quantified at 24 h using the crystal violet assay at a wavelength of 600 nm. Data represent mean ± standard deviation (SD) from three independent experiments performed in triplicate. Statistical significance was determined by ANOVA with Tukey’s post hoc test (* *p* < 0.05, ** *p* < 0.01, *** *p* < 0.001). Results from mixed-effects model analysis are provided in the Supplementary Materials (Figures S1 and S2).

MVs were added to *S. mutans* cultures in a dose-dependent manner and compared with controls lacking MVs. Following supplementation with 1, 2, 5, 10, 15 and 20 μg/mL of MVs in 1% BHIs for 24 h, biofilm biomass was quantified using the CV assay. Concentrations ≥ 5 μg/mL significantly enhanced biofilm formation (*p* < 0.05) (Figure 2a).

To determine the time point at which MVs exert their effect, 5 μg/mL MVs were added at 0, 6, or 12 h during biofilm development. The results showed that biofilm formation was enhanced when MVs were added at 0 or 6 h, but no significant effect was observed when MVs were added at 12 or later (Figure 2b).

**Figure 3:**
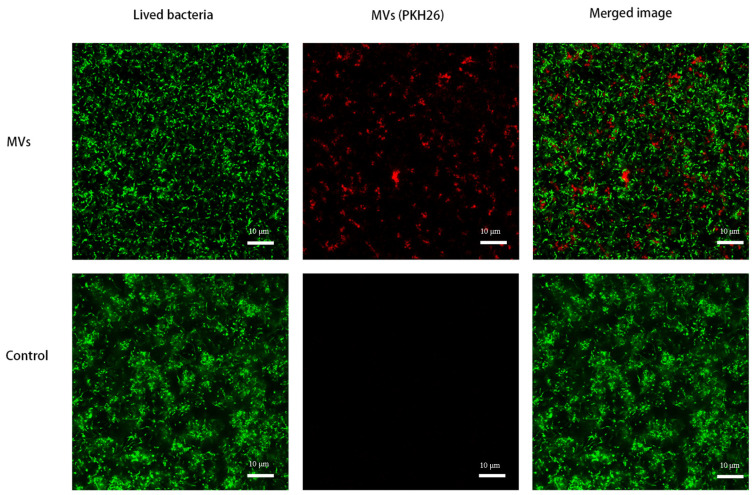
Localization of MVs within *S. mutans* biofilms. MVs derived from *S. mutans* were labeled with PKH26 (red) and added to *S. mutans* cultures in 1% BHI for 24 h. Confocal laser scanning microscopy (20× magnification) revealed incorporation of labeled MVs (red) within the biofilm matrix of *S. mutans* (green); bar = 10 μm.

MVs were labeled with PKH26 and added to *S. mutans* in 1% BHI. Confocal micrographs showed that the labeled MVs were incorporated into the *S. mutans* biofilm and were present within the biofilm matrix (Figure 3).

### 3.3. Evaluation of the Effect of MVs on S. mutans Biofilm Formation

**Figure 4:**
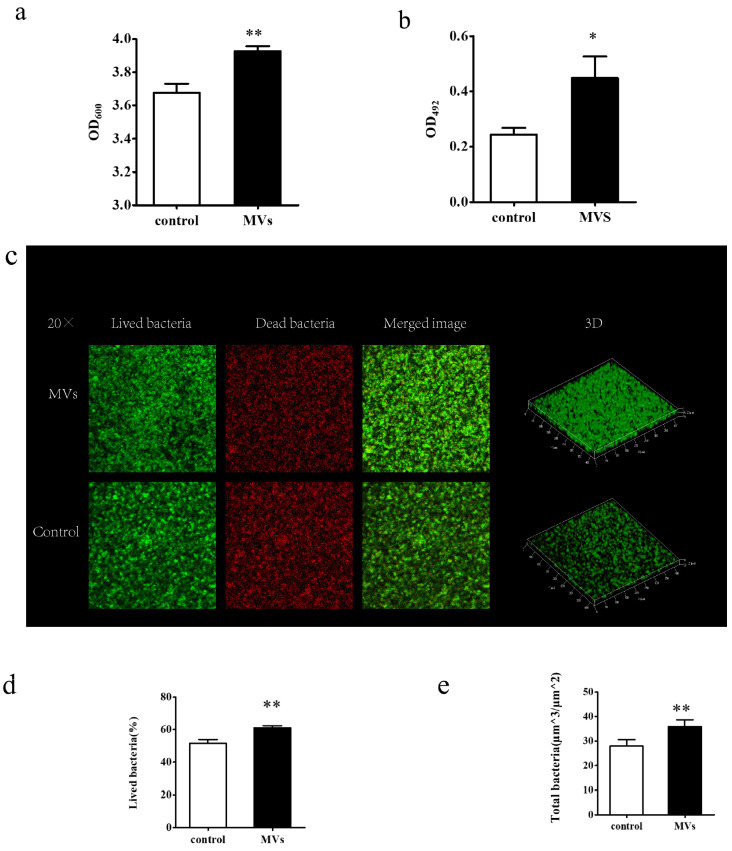
Effect of MVs on *S. mutans* biofilm formation. *S. mutans* cultured in 1% BHI was treated with PBS (control) or MVs for 24 h. (**a**) Biofilm biomass was evaluated by the CV assay. (**b**) Biofilm metabolic activity was evaluated by XTT assay. (**c**) Representative CLSM images showing live cells (green), dead cells (red), merged channels, and three-dimensional reconstructions of 24 h biofilms. (**d**,**e**) Quantitative analysis of the proportion of viable bacteria and total biofilm biomass. Data are presented as the mean ± SD (* *p* < 0.05, ** *p* < 0.01).

Both CV and XTT assays were conducted to evaluate changes in *S. mutans* biofilm characteristics in response to MVs. The CV assay showed that MVs significantly increased biofilm biomass (*p* < 0.05) (Figure 4a). Consistently, the XTT assay revealed higher metabolic activity in the MV-treated biofilms compared to controls (*p* < 0.05) (Figure 4b).

The effect of MVs on bacterial viability within biofilms was further evaluated by CLSM using LIVE/DEAD staining. In the images, green fluorescence indicates viable cells, and red fluorescence indicates dead cells. MV-treated biofilms exhibited a higher density of green-stained bacteria relative to the control group, indicating enhanced biofilm biomass (Figure 4c). Quantitative analysis confirmed an increased ratio of viable cells (Figure 4d) and greater total biofilm biomass (Figure 4e) in the presence of MVs.

**Figure 5:**
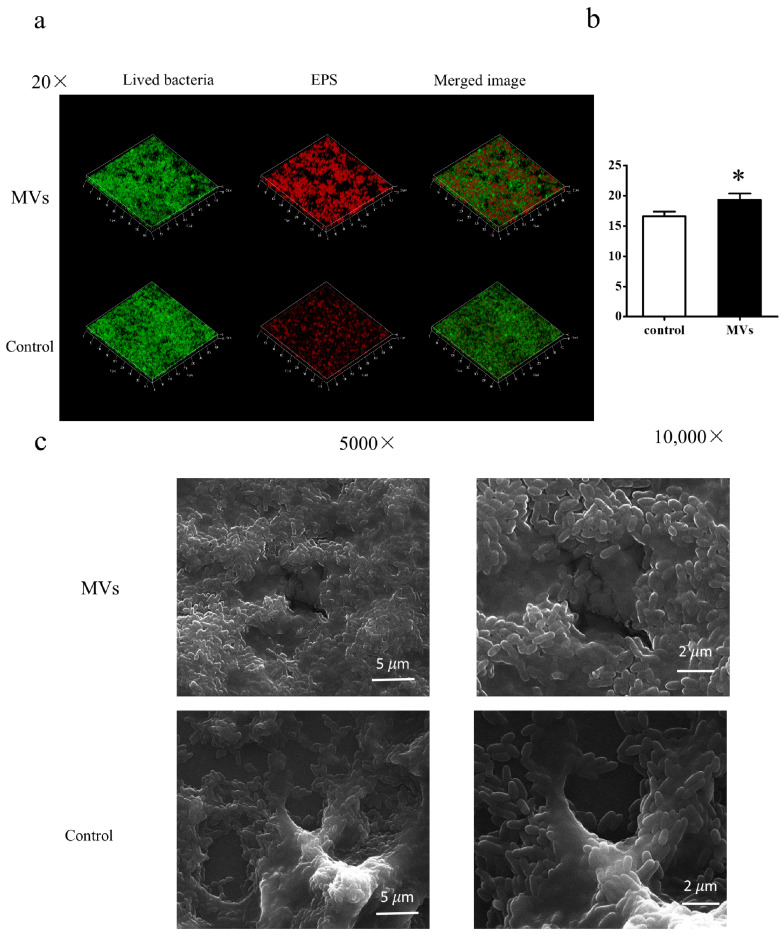
Evaluation of EPS in *S. mutans* biofilms. *S. mutans* cultured in 1% BHI was treated with PBS (control) or MVs for 24 h. (**a**) Representative CLSM images of 24 h biofilms. Bacterial cells were labeled with SYTO9 (green), and EPS with Alexa Fluor 647 (red). Images were obtained at 20× magnification. (**b**) Quantification of EPS biomass from 5 randomly selected regions per sample, with experiments repeated three times. Data are presented as mean ± SD (* *p* < 0.05). Results from mixed-effects model analysis are provided in the Supplementary Materials (Figure S3). (**c**) Representative SEM images of 24 h biofilms at 5000× and 10,000× magnification, showing structural differences between control and MV-treated biofilms.

Moreover, three-dimensional CLSM reconstruction of *S. mutans* biofilms confirmed that EPS production increased following MV treatment (*p* < 0.05) (Figure 5a,b). SEM analysis further revealed structural differences between biofilms formed without MVs (control) and those supplemented with MVs (Figure 5c). In the presence of MVs, biofilms exhibited a dense matrix with bacterial cells embedded on the surface, suggesting an increase in overall biomass. This observation is supported by quantitative CLSM data, which showed significantly higher total bacterial biovolume (Figure 4e) and EPS biovolume (Figure 5b) in MV-treated biofilms. Additionally, MVs were consistently observed surrounding bacterial cells in all biofilm images treated with MVs.

### 3.4. Effect of MVs on the mRNA Levels of S. mutans Biofilm Virulence Factors

**Figure 6:**
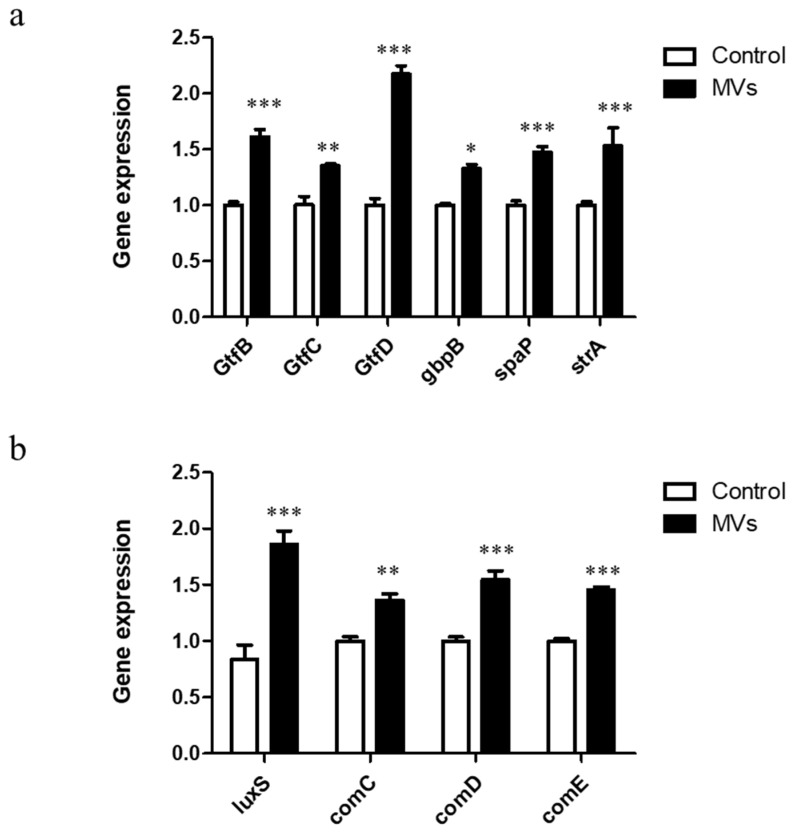
Effect of MVs on the expression of *S. mutans* biofilm virulence genes. qRT-PCR analysis of 24 h *S. mutans* biofilms treated with PBS (control) or MVs. (**a**) Expression of adhesion-related genes (*srtA*, *spaP*, *gtfB*, *gtfC*, *gtfD*, *gbpB*). (**b**) Expression of quorum-sensing-related genes (*luxS*, *comC*, *comD*). Data are presented as mean ± SD (*n* = 3; * *p* < 0.05, ** *p* < 0.01, *** *p* < 0.001).

The impact of MVs on the expression of adhesion-related genes was assessed, including both sucrose-independent and sucrose-dependent adhesion factors. In biofilms treated with MVs, the expression levels of *srtA* and *spaP* increased by approximately 1.53- and 1.47-fold, respectively, compared with untreated biofilms. Expression of sucrose-dependent adhesion genes, including *gtfB*, *gtfC*, *gtfD*, and *gbpB*, was significantly increased by 1.61-, 1.35-, 2.17- and 1.20-fold, respectively (Figure 6a). Similarly, MV treatment enhanced the transcription of quorum-sensing-related genes. Expression levels of *luxS*, *comC*, and *comD* increased by 1.86-, 1.36- and 1.55-fold, respectively, compared with controls (Figure 6b). These results indicate that MVs can promote biofilm formation by upregulating genes involved in adhesion and quorum-sensing pathways.

## 4. Discussion

*S. mutans* is a major etiological factor of dental caries, forming biofilms known as dental plaque that contribute to tooth decay. In this study, we provide detailed evidence that MVs derived from *S. mutans* enhance biofilm information.

We successfully isolated MVs from *S. mutans* and confirmed their bilayered, cup-shaped morphology, with an average diameter of approximately 100 nm. These characteristics are consistent with MVs derived from other Gram-positive bacteria [12,29].

Our results demonstrate that supplementation with MVs enhances *S. mutans* biofilm formation in a dose-dependent manner. CV assays showed that concentrations of ≥5 μg/mL MVs significantly increased biofilm biomass. These findings are consistent with previous studies, such as Fulsundar et al., who reported that 6, 12 and 18 µg/mL MVs increased 24 h biofilm formation by 0.69, 1.75 and 2.68 fold, respectively [28]. The observation that a threshold concentration (5 μg/mL) was required suggests that a critical amount of MVs may be necessary to achieve sufficient incorporation into the biofilm matrix or to trigger downstream signaling pathways. Notably, the effect of MVs was observed only during the early stages of biofilm development (0–6 h); addition at 12 h or later did not alter biofilm formation. This suggests that MVs may serve as a structural framework for the developing matrix, with their function diminishing at later stages. Similarly, a recent review by Jeong et al. (2024) highlighted the dual role of bacterial extracellular vesicles in biofilm formation—they can both promote early-stage biofilm development and disperse mature biofilms, depending on the context [30]. Our time-course data support the notion that the promoting effect is most critical in the initial adhesive phase when the extracellular matrix is being assembled.

We further investigated MVs as a structural component of *S. mutans* biofilms. Biofilms are complex, sessile microbial communities embedded in an extracellular matrix [31]. PKH26-labeled MVs were observed within the biofilm matrix, confirming that MVs are a component of the biofilm and can function within the matrix. EPS, a critical component for mechanical stability, adhesion, and three-dimensional biofilm structure [32], was significantly increased following MV treatment. CLSM analysis revealed that higher biomass increased total bacterial numbers and a greater proportion of viable cells. SEM images showed that MVs filled gaps in bacterial cells, suggesting they facilitate intercellular adhesion and biofilm growth, consistent with observations in *Acinetobacter radioresistens* biofilms treated with MVs [28]. These observations are supported by recent findings from Zhao et al. (2025), who demonstrated that alterations in membrane vesicle production affect eDNA content and biofilm integrity in *S. mutans* [33]. These results imply that MVs contribute to early-stage biofilm development.

Adhesion in *S. mutans* biofilms within dental plaque occurs via sucrose-independent and sucrose-dependent mechanisms. Sucrose-independent adhesion-related virulence genes (*srtA* and *spaP*) and sucrose-dependent adhesion-related virulence genes (*gtfs* and *gbps*) play crucial roles in biofilm formation. In this study, the expression of *srtA* and *spaP* was increased in the MV-treated group. *S. mutans* possesses three GTFs encoded by *gtfB*, *gtfC*, and *gtfD*, all of which showed significantly higher expression following MV supplementation. In addition, *gbpB*, which contributes to biofilm development, was also upregulated.

Quorum-sensing (QS) systems, including the LuxS system and ComCDE system, are also critical for *S. mutans* biofilm formation. The LuxS system catalyzes the formation of the signal peptide AI-2, mediating intra- and interspecies interactions within multispecies plaque communities [34]. The ComCDE system regulates cell–cell communication and modulates the expression of *gtfB*, *gtfC*, and *gtfD* [35,36,37]. In this study, both the LuxS and ComCDE systems showed increased expression in the presence of MVs. Recent work by Nagasawa et al. (2025) has provided new insights into the link between quorum sensing and MV production, demonstrating that the Com system regulates MV release through LytF-dependent autolysis in a subpopulation of cells [38]. This suggests a potential feedback loop: MVs may influence QS gene expression, and QS in turn may regulate MV biogenesis. Upregulation of these virulence genes likely contributes to higher numbers of viable bacteria and increased EPS biomass in the biofilm. Biofilm formation in *S. mutans* is complex, and additional regulatory systems (such as the VicRK and CovR systems) are likely involved, warranting further investigation.

As bacterial MVs are nanoscale particles, their surface charge (zeta potential) may influence biological functions by governing electrostatic interactions with negatively charged bacterial surfaces and the extracellular matrix [39]. Recent studies using engineered nanoparticles further support this notion. For instance, Aragão et al. (2025) developed chitosan nanoparticles loaded with epigallocatechin-3-gallate and demonstrated that these nanoparticles, with a positive zeta potential of approximately +30 mV, effectively disassembled preformed *S. mutans* biofilms, likely due to enhanced electrostatic attraction to the negatively charged biofilm matrix [40]. Similarly, Krishnasamy et al. (2024) synthesized platinum nanoparticles derived from Desmostachya bipinnata and reported that these nanoparticles, with a negative zeta potential of −19.3 mV, exhibited robust antibiofilm activity, reducing biofilm formation by up to 70% and significantly downregulating the expression of *gtfB*, a key gene involved in EPS synthesis [41]. Moreover, Panda et al. (2024) developed nanoliposomes loaded with improved Toluidine Blue O, which carried a negative surface charge of approximately −39.54 mV and served as effective nanocarriers against *S. mutans* biofilms, highlighting the critical role of surface charge in nanoparticle–biofilm interactions [42]. Collectively, these findings underscore that the surface charge of nanoscale particles—whether positive or negative—can profoundly influence their interactions with biofilms, affecting both biofilm structure and microbial physiology.

Although we successfully characterized the size distribution of *S. mutans* MVs in this study, we did not measure their zeta potential. Given the established importance of surface charge in nanoparticle–biofilm interactions, this represents an important avenue for future investigation. Zeta potential is a critical determinant of colloidal stability, membrane adhesion, and cellular uptake, all of which are likely to influence how MVs interact with bacterial surfaces and the biofilm matrix. Future studies should systematically characterize the surface charge of *S. mutans* MVs under different growth conditions (e.g., pH, nutrient availability, or oxygen tension), as these environmental factors are known to modulate bacterial membrane composition and vesicle biogenesis, potentially leading to significant variations in MV surface properties. Moreover, beyond simple characterization, it will be essential to investigate whether MVs with varying surface charges differentially affect biofilm formation—for instance, by comparing the biofilm-promoting activity of MVs generated under distinct physicochemical conditions or following chemical modification of their surface charge. Such studies could elucidate whether electrostatic interactions are a key mechanism by which MVs promote biofilm development. Ultimately, this line of inquiry will provide deeper insights into the functional roles of MVs in bacterial physiology and pathogenesis, potentially opening new avenues for targeting MVs in anti-biofilm strategies.

## 5. Conclusions

*S. muants* MVs function as integral components of biofilms, enhancing early-stage biofilm formation. These findings expand our understanding of *S. mutans* MVs and suggest they may play an important role in caries pathogenesis research.

## Figures and Tables

**Table 1:** Nucleotide sequences of primers used for qRT-PCR.

Gene	Forward	Reverse
*gtfB*	ACACTTTCGGGTGGCTTG	GCTTAGATGTCACTTCGGTTG
*gtfC*	CCAAAATGGTATTATGGCTGTCG	GAGTCTCTATCAAAGTAACGCAGT
*gtfD*	TTGACGGTGTTCGTGTTGAT	AAAGCGATAGGCGCAGTTTA
*gbpB*	AGCAACAGAAGCACAACCATCAG	CCACCATTACCCCAGTAGTTTCC
*srtA*	GAAGCTTCCTGTAATTGGCG	TTCATCGTTCCAGCACCATA
*spaP*	GACTTTGGTAATGGTTATGCATCAA	TTTGTATCAGCCGGATCAAGTG
*comC*	GACTTTAAAGAAATTAAGACTG	AAGCTTGTGTAAAACTTCTGT
*comD*	CTCTGATTGACCATTCTTCTGG	CATTCTGAGTTTATGCCCCTC
*comE* *LuxS*	CCTGAAAAGGGCAATCACCAG CCAGGGACATCTTTCCATGAGAT	GGGGCATAAACTCAGAATGTGTCG ACGGGATGATTGACTGTTCCC
*16sRNA*	CTTACCAGGTCTTGACATCCCG	ACCCAACATCTCACGACACGAG

## Data Availability

The original contributions presented in this study are included in the article/Supplementary Material. Further inquiries can be directed to the corresponding author.

## Supplementary Materials

The following supporting information can be downloaded at: https://www.mdpi.com/article/10.3390/microorganisms14040826/s1, Figure S1: Mixed-effects model results for Figure 2a (dose-dependent effect of MVs); Figure S2: Mixed-effects model results for Figure 2b (time-dependent effect of MVs); Figure S3: Mixed-effects model results for Figure 5b (EPS biovolume).
