# Supplementary material for: Membrane Vesicles Improve Streptococcus mutans Early Biofilm Formation
Source: Microorganisms. 2026 Apr 3;14(4):826. doi: 10.3390/microorganisms14040826 (PMC13119075; doi:10.3390/microorganisms14040826)
Supplement: Supplementary file 1 [file microorganisms-14-00826-s001.zip › microorganisms-4193983-supplementary.pdf]

Supplementary Materials

Figure S1. Mixed-effects model results for Figure 2a (dose-dependent effect of MVs)

(A) Fixed effects table

Source	*P* value	Summary	*F* (*DFn*, *DFd*)
Treatment	<0.0001	****	*F* (6, 42) = 13.61
Experiment	0.9735	ns	*F* (1.611, 33.83) = 0.01271
Treatment × Experiment	0.9988	ns	*F* (12, 42) = 0.1763

Mixed-effects model with experiment as random effect and treatment as fixed effect.

(B) Multiple comparisons (Dunnett's test vs. Control)

Comparison	Mean difference	95% CI	Adjusted *P* value
control vs. 1μg/ml	-0.001111	-0.1310 to 0.1288	>0.9999
control vs. 2μg/ml	-0.03367	-0.09252 to 0.02518	0.4029
control vs. 5μg/ml	-0.1423	-0.2210 to -0.06369	0.0008
control vs. 10μg/ml	-0.1807	-0.2203 to -0.1410	<0.0001
control vs. 15μg/ml	-0.1476	-0.1912 to -0.1039	<0.0001
control vs. 20μg/ml	-0.1862	-0.1310 to 0.1288	<0.0001

Comparison Mean difference 95% CI Adjusted *P* value

(C) Corresponding graph

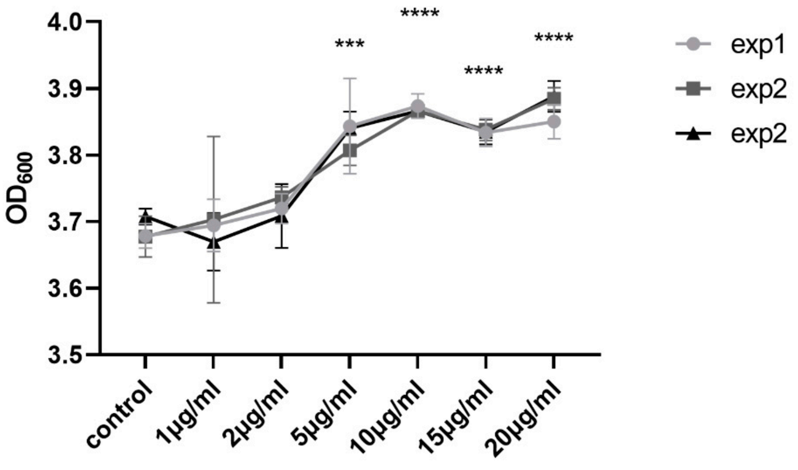

Mixed-effects model analysis of the dose-dependent effect of MVs on *S. mutans* biofilm formation (Figure 2a). (A) Fixed-effects table showing the effects of treatment (MV concentration), experiment (biological replicate), and their interaction. ****, *P*<0.0001; ns, not significant. (B) Dunnett's multiple comparisons test comparing each MV concentration to the control group. Data are from three independent experiments, each with three technical replicates. Mixed-effects model was fitted with experiment as

random effect and treatment as fixed effect. (C) Corresponding graph. ***, *P*<0.001; ****, *P*<0.0001.

Figure S2. Mixed-effects model results for Figure 2b (time-dependent effect of MVs)

(A) Fixed effects table

Source	*P* value	Summary	*F* (*DFn*, *DFd*)
Treatment	<0.0001	****	F (3, 24) = 34.12
Experiment	0.7139	ns	F (1.394, 16.72) = 0.2337
Treatment × Experiment	0.9160	ns	F (6, 24) = 0.3272

Mixed-effects model with experiment as random effect and treatment as fixed effect.

(B) Multiple comparisons (Dunnett's test vs. Control)

Comparison	Mean difference	95% CI	Adjusted *P* value
Control vs. 0h+MVs	-0.2483	-0.2942 to -0.2025	<0.0001
Control vs. 6h+MVs	-0.1224	-0.1797 to -0.06522	0.0001
Control vs. 12h+MVs	-0.05856	-0.1302 to 0.01307	0.1190

(C) Corresponding graph

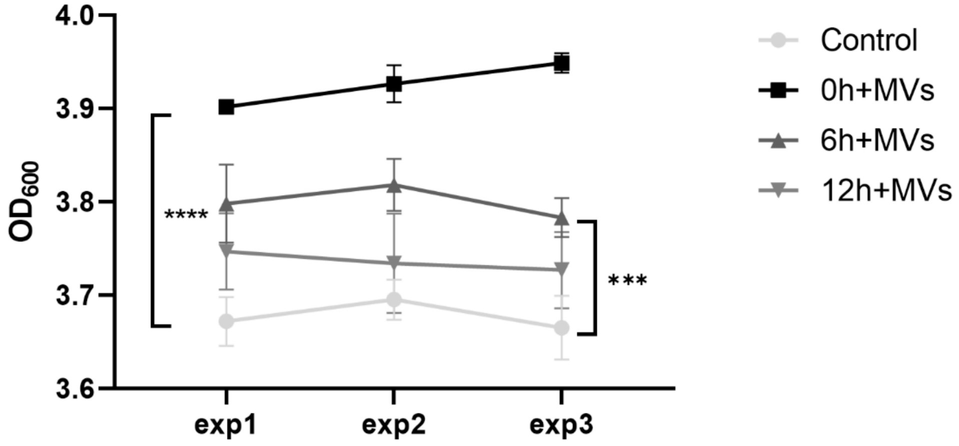

Mixed-effects model analysis of the time-dependent effect of MVs on *S. mutans* biofilm formation (Figure 2b). (A) Fixed-effects table. ****, *P*<0.0001; ns, not significant (B) Dunnett's multiple comparisons test comparing each time point to the control group. Mixed-effects model was fitted with experiment as random effect and treatment as fixed effect. (C) Corresponding graph. ***, *P*<0.001; ****, *P*<0.0001.

Figure S3. Mixed-effects model results for Figure 5b (EPS biovolume)

(A) Fixed effects table

Source	*P* value	Summary	*F* (*DFn*, *DFd*)
Treatment	<0.0001	****	*F* (1, 24) = 191.6
Experiment	0.7473	ns	*F* (1.553, 18.63) = 0.2222
Treatment × Experiment	0.0752	ns	*F* (2, 24) = 2.889

Mixed-effects model with experiment as random effect and treatment as fixed effect.

(B) Corresponding graph

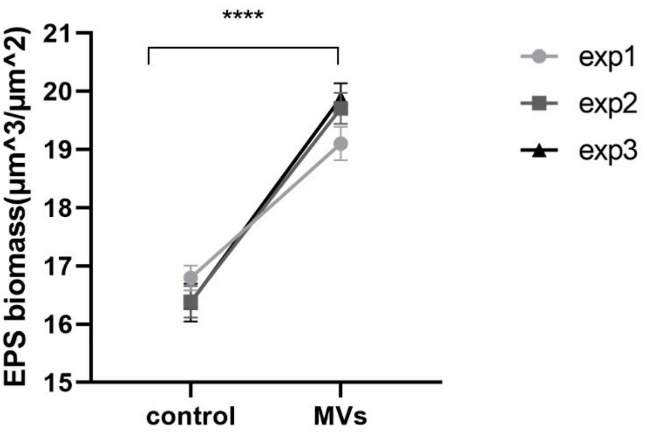

(A) Fixed effects table. ****, *P*<0.0001; ns, not significant. (B) Corresponding graph. ****, *P*<0.0001. Mixed-effects model analysis of EPS biomass in *S. mutans* biofilms (corresponding to Figure 5b in the main text). The treatment effect (MV vs. Control) was highly significant ($P < 0.0001$), confirming that MV treatment significantly increased EPS production. The experiment effect was not significant, indicating good reproducibility across the three independent experiments. Mixed-effects model was fitted with experiment as random effect and treatment as fixed effect.
